# Bioactive Compounds of Aboveground Organs *Salicornia europaea* L. of East Kazakhstan Flora

**DOI:** 10.3390/molecules31162778

**Published:** 2026-08-10

**Authors:** Laura Kazhygeldiyeva, Lazzyat Orazzhanova, Binur Mussabayeva, Alfira Sabitova, Batiyash Silybayeva, Akmaral Issayeva

**Affiliations:** 1 Department of Chemistry and Ecology, Research School of Physical and Chemical Sciences, Shakarim University, Glinky Str., 20A, Semey 071412, Kazakhstan; lauka_nurik2014@mail.ru (L.K.); lazzyat.orazzhanova.70@mail.ru (L.O.); alfa-1983@mail.ru (A.S.); akmaral.issayeva@bk.ru (A.I.); 2 Department of Natural Sciences, Pedagogical Institute, Astana International University, Kabanbay Batyr Ave, 8, Astana 010017, Kazakhstan; 3 Department of Applied Biology, Faculty of Information Technologies and Economics, Alikhan Bokeikhan University, Mangilik el Str., 11, Semey 070000, Kazakhstan; batiyashsilybaeva@mail.ru; 4 Research Institute of Ecology and Biotechnology, Shymkent University, Zhibek zholy Str., 6, Shymkent 160031, Kazakhstan

**Keywords:** chemical composition, *Salicornia europaea* L., flavonoids, phenolic compounds, antioxidant activity, vitamins

## Abstract

This study presents the phytochemical profile and antioxidant activity of the aboveground organs of *Salicornia europaea* L. collected from a natural population in East Kazakhstan. The chemical composition of the plant sample was studied using a complex of modern analytical methods, including HPLC, GC-MS, IR-Fourier spectroscopy, and elemental analysis. Metabolites were putatively annotated based on mass spectrometry data, corresponding to MSI level 2. It was found that the content of flavonoids was 2.40 ± 0.02 mg QE/g of dry raw materials, and the content of polyphenols was 6.73 ± 0.03 mg GAE/g. The antioxidant activity (ABTS test) reached 7.85 ± 0.04 mg TE/g. The concentration of fat-soluble and water-soluble vitamins was C—1.27 ± 0.12 mg/100 g, A—1.16 ± 0.11 mg/100 g and E—3.89 ± 0.38 mg/100 g. The IR characterization of plant raw materials and ash was carried out, the indicators of the elemental composition (TC, TOC, TIC, TN, TS) were determined. The totality of the obtained data indicates the phytochemical potential of *Salicornia europaea* L. growing in the territory of East Kazakhstan and may serve as a basis for further applied research.

## 1. Introduction

In the modern pharmaceutical and cosmetic industry, there is an increasing interest in biologically active substances (BAS) of plant origin due to their structural diversity, multidirectional action and biocompatibility [1,2]. Plants adapted to extreme environmental conditions, including halophytes, accumulate high concentrations of secondary metabolites (phenolic acids, flavonoids, terpenoids, etc.), which makes them promising raw materials for the production of natural antioxidants [3,4].

**Figure 1:**
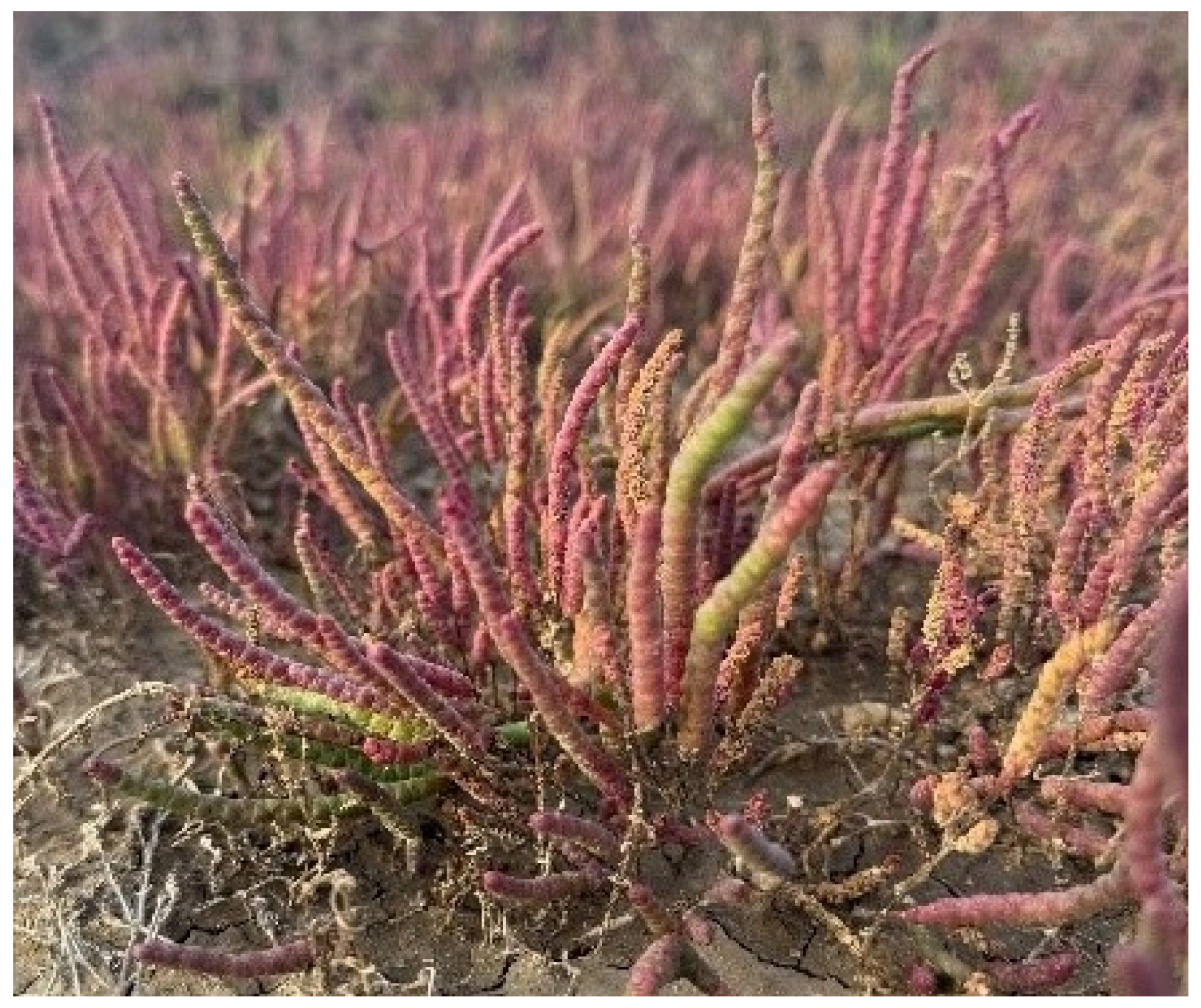
*Salicornia europaea* L.

*Salicornia europaea* L. is an annual halophyte belonging to the genus *Salicornia* and the family *Amaranthaceae* (formerly part of the *Chenopodiaceae* family) [5,6]. It is a low-growing shrub 10–40 cm high with jointed shoots [7,8] (Figure 1).

This species is widespread in Europe, Asia, and North Africa and occurs in the salt marshes of the Republic of Kazakhstan [9,10].

The phytochemical composition of plants of the genus *Salicornia* has repeatedly been the subject of review studies [11,12,13]. In the study by Kim et al. [14], 89 individual compounds belonging to various chemical classes were described for this species: oleanane triterpenoid saponins, caffeoylquinic acid derivatives, flavonoids, chromones, sterols, lignans, and aliphatic compounds. This chemical diversity indicates that the biological activity of *S. europaea* cannot be explained by the action of only one group of substances and is probably formed due to the complex contribution of phenolic, terpene, sterol and other components. Of particular significance are caffeoylquinic acid derivatives (3,5-dicaffeoylquinic acid, methyl-3,5-dicaffeoylquinate, 3,4-dicaffeoylquinic acid), which exhibit pronounced antioxidant activity in the DPPH assay and inhibit the formation of cholesteryl-ester hydroperoxides. From a chemical standpoint, this is attributed to the presence of caffeoyl moieties and catechol hydroxyl groups, which are capable of participating in electron-transfer or hydrogen-atom-transfer reactions and stabilizing free-radical species.

The review by Limongelli et al. [11] systematized data on the polyphenolic composition of plants of the genus *Salicornia*. Chlorogenic, gallic, protocatechic, caffeic, p-coumaric, and ferulic acids, as well as quercetin-3-O-β-D-glucopyranoside, isorhamnetin-3-O-β-D-glucopyranoside, quercetin, and isoramnetin, have been identified in *S. europaea* extracts. The flavonoid fraction includes quercetin, isoramnetin and their glycosides.

Using GC–MS and LC–MS/MS, fatty acids were identified, among which oleic acid (36.55%) [1], α-tocopherol (2.1 ± 0.3 mg/100 g) [15] dominated, as well as more than 90 bioactive compounds related to saponins, flavonoids, phenols, chlorogenic acids, terpenoids, sterols, lignans, aldehydes, tocopherols, etc. [15].

The broad spectrum of biological activities of *S. europaea*—including antioxidant, anti-inflammatory [16], antibacterial [17], and immunomodulatory [14,18,19,20,21]—is attributed precisely to this chemical diversity.

The high salt tolerance of *S. europaea* is associated with a complex set of physiological and biochemical adaptive mechanisms, including ion accumulation, osmotic regulation, and activation of antioxidant defense [22,23]. Under salt stress, *S. europaea* exhibits changes in metabolic pathways related to phenylpropanoid metabolism, flavone and flavonol biosynthesis, as well as the accumulation of stress-associated metabolites, including proline and quercetin and kaempferol derivatives [23]. These processes may influence the composition of secondary metabolites and contribute to the accumulation of compounds with antioxidant potential, thereby supporting the investigation of polyphenols, flavonoids, vitamins, and antioxidant activity in the aerial parts of *S. europaea* [14,20].

The territory of Kazakhstan, characterized by an arid climate, high soil salinity and water scarcity, creates favorable conditions for the growth of *S. europaea* over extensive areas. However, despite the widespread distribution, pronounced bioactive properties and high ecological plasticity, studies of the phytochemical composition of Kazakhstani populations of *S. europaea* remain fragmentary and practically undisclosed to date. The available studies by domestic researchers are primarily focused on the applied and technological aspects of its utilization. Therefore, Kaisarova et al. [24] showed the possibility of using *Salicornia* from the Turkestan region as a partial substitute for table salt. Aubakirova et al. [25] considered *Salicornia* as a promising component of marine aquaponics [24,25].

The sharply continental climate of East Kazakhstan, together with the presence of saline soils and solonchak habitats in the areas where *S. europaea* grows, may influence the phytochemical profile of the plant, including the qualitative composition and quantitative content of biologically active compounds.

Data on the chemical composition of *S. europaea*, which grows in Eastern Kazakhstan, are presented in the work of Mussabayeva et al. [26], where phenolic and volatile compounds were identified. However, comprehensive data on the phytochemical features and antioxidant potential of the aerial parts of this species remain limited.

Therefore, the aim of the present study was to expand the existing data on the chemical composition of *S. europaea* collected from a saline habitat in Eastern Kazakhstan by conducting a comprehensive analysis of its aerial parts using elemental analysis (C, N, S), GC-MS, FTIR spectroscopy, spectrophotometric determination of polyphenols, flavonoids, and vitamins (A, C, E), as well as evaluation of antioxidant activity using the ABTS assay.

The obtained data provide additional phytochemical information on *S. europaea* collected from a saline habitat in Eastern Kazakhstan and may serve as a basis for further studies of its biological potential.

## 2. Results

**Figure 2:**
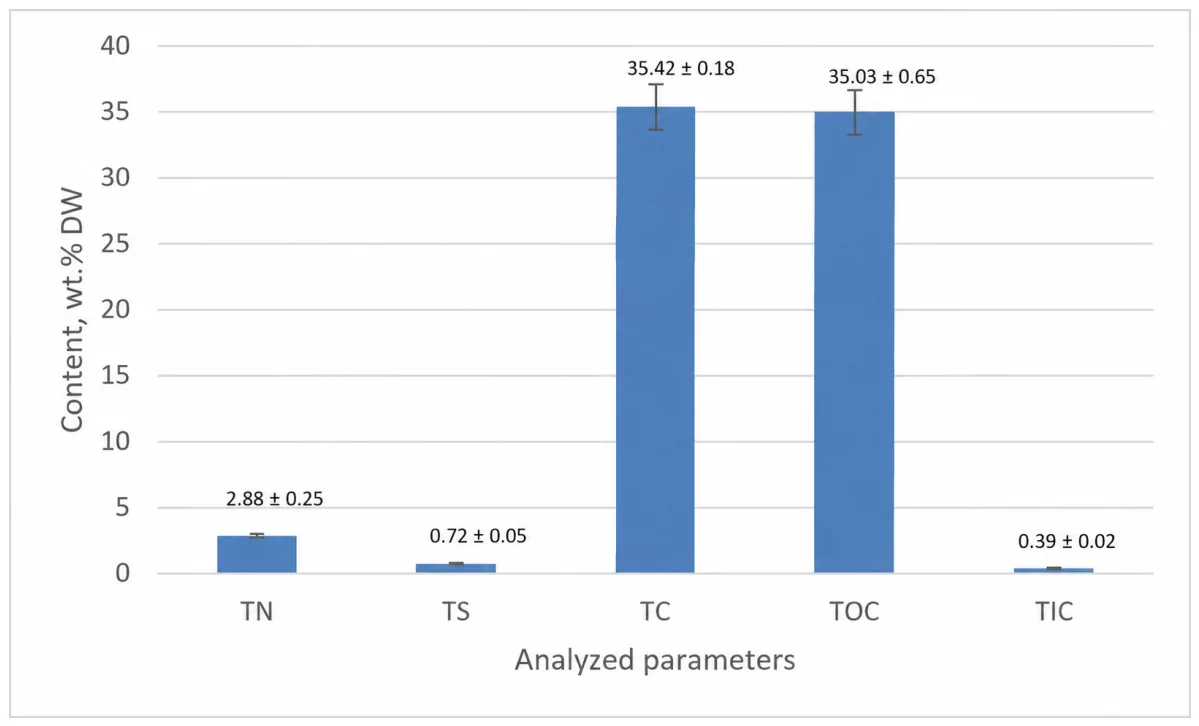
Contents of total nitrogen (TN), total sulfur (TS), total carbon (TC), total organic carbon (TOC), and total inorganic carbon (TIC) in the aboveground organs of *S. europaea*. Data are presented as mean ± SD (*n* = 3). Error bars indicate SD.

In our previous study [27], we reported data on the content of total carbon (TC), organic carbon (TOC), inorganic carbon (TIC), and total nitrogen (TN) and sulfur (TS) in *S. europaea* samples growing in Eastern Kazakhstan. However, their detailed characteristics were not given. This paper provides an interpretation of the previously obtained results (Figure 2).

**Figure 3:**
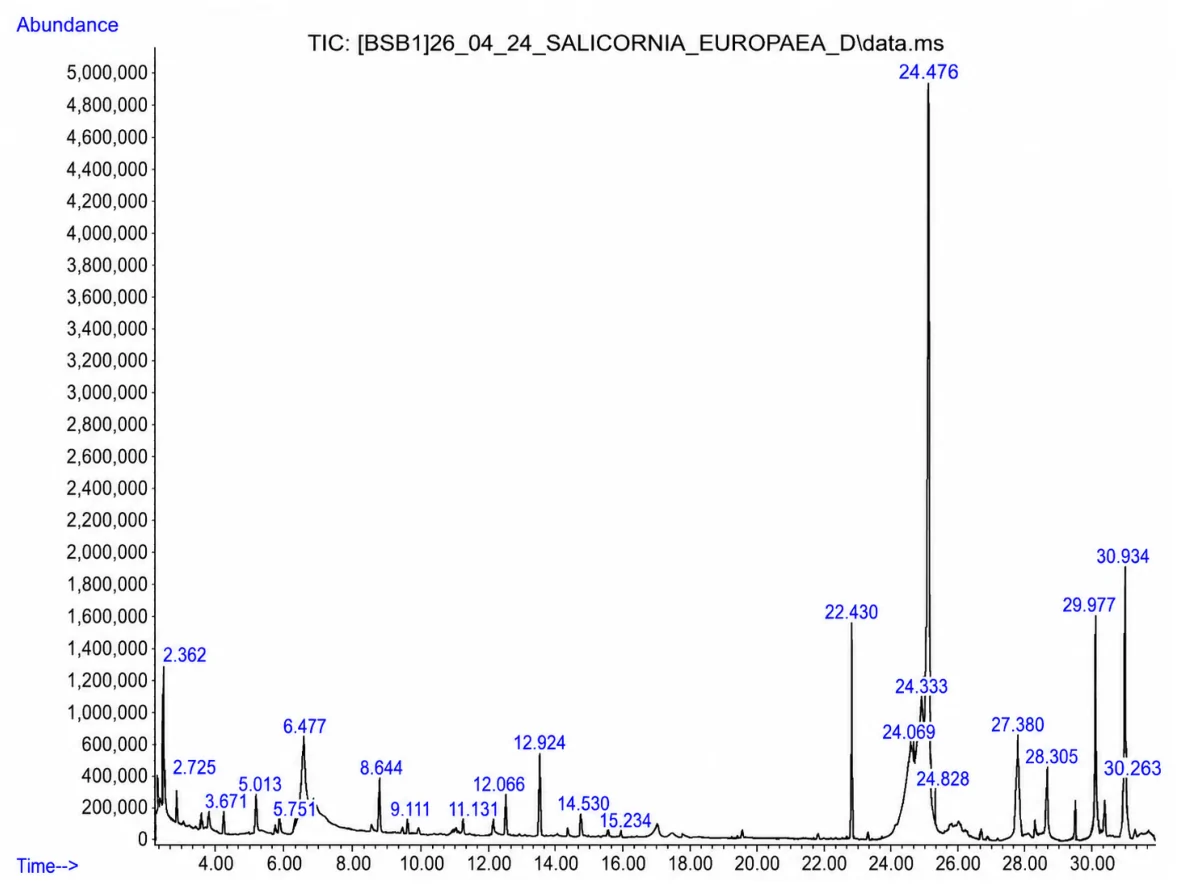
Chromatogram of *S. europaea* ethanol extract.

Based on GC-MS analysis, several compounds were tentatively annotated in the ethanolic extract of *S. europaea* (Figure 3, Table 1).

**Figure 4:**
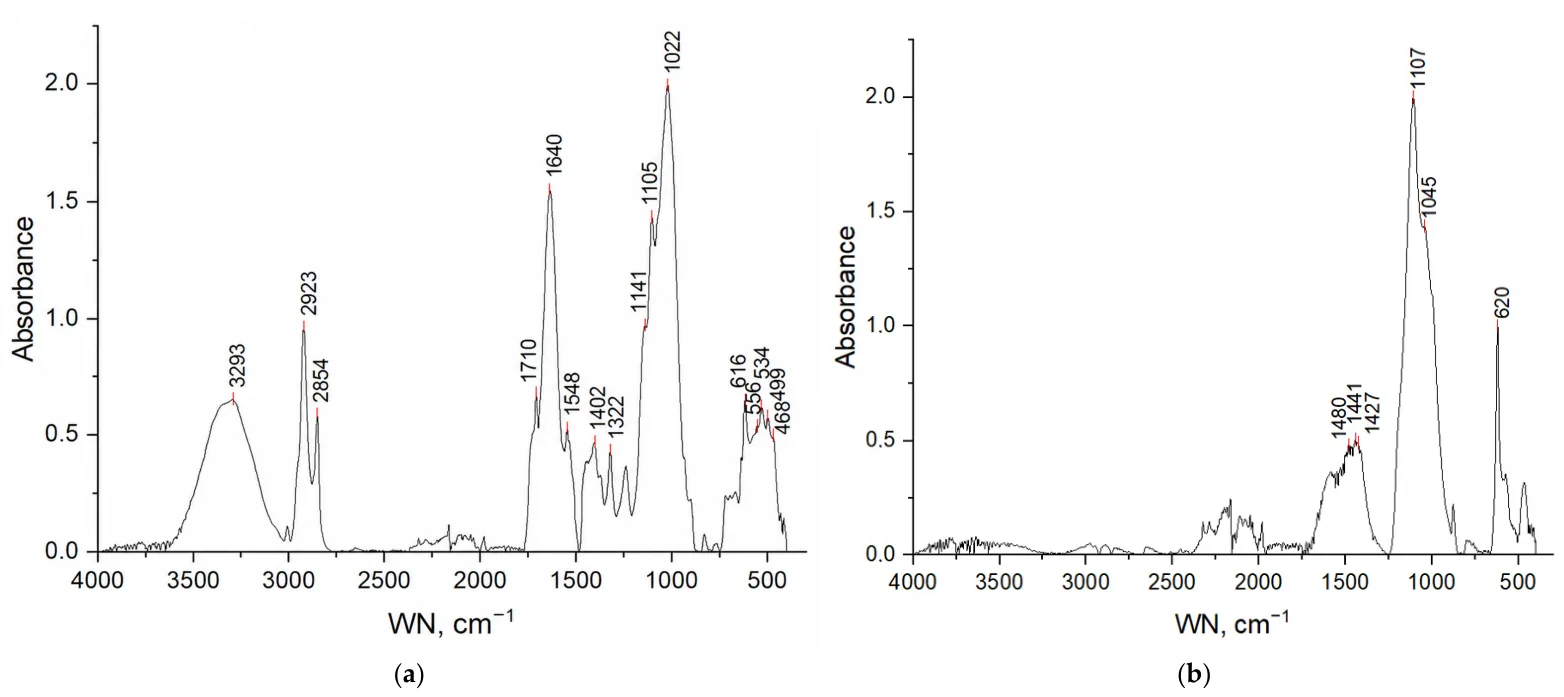
IR spectra of the crude sample (**a**) and the ash fraction (**b**) of *S. europaea*.

Fourier transform infrared spectroscopy of the crude sample and the ash fraction of the studied plant sample (Figure 4, Table 2) made it possible to identify the functional groups of *S. europaea* adapted to the conditions of Eastern Kazakhstan. Figure 4a,b show the IR spectra of the raw sample and the ash fraction of *S. europaea*.

The results of ultra-performance liquid chromatography with mass spectrometric detection (UPLC-HRMS/MS) indicate the presence of a wide range of secondary metabolites in the plant raw materials of *S. europaea*. As a result of the analysis, 18 key secondary metabolites were annotated, with their structures characterized based on accurate *m*/*z* values and fragmentation patterns (Table 3).

The studied sample of *S. europaea* is characterized by the presence of phenolic compounds and flavonoids. Thus, the total content of polyphenols was 6.73 ± 0.03 mg GAE/g, and the total content of flavonoids was 2.40 ± 0.02 mg QE/g of dry raw materials. The value of antioxidant activity according to the ABTS method was 7.85 ± 0.04 mg TE/g, which indicates the presence of compounds with antioxidant potential.

The present study provides information on the content of fat-soluble vitamins in *S. europaea* growing in Eastern Kazakhstan. The results are presented in Table 4.

## 3. Discussion

An analysis of the elemental composition of *S. europaea* showed a predominance of organic matrix (TOC 35.03 ± 0.65%), while the proportion of inorganic carbon is insignificant (TIC 0.39 ± 0.02%). This indicates a high proportion of carbonaceous metabolites in the biomass of aboveground organs and a small contribution of mineral carbon compounds to the structure of shoots (absence of carbonate inclusions), which is typical for halophytes growing in soils with a neutral or slightly alkaline environment [41]. This may facilitate the release of biologically active substances.

The TN content was 2.88 ± 0.25%, which significantly exceeds the values given for vegetative organs of *S. europaea* from Korean populations (1.22–1.78%) [41], as well as for plants grown under controlled salinity conditions (0.41–0.58%) [42]. The high TN content, as well as the ratio C/N = 12.3 in the East Kazakhstan sample, indicates the active synthesis of nitrogen-containing osmolytes in response to osmotic stress caused by high concentrations of chlorides and sulfates in the soil [43].

An increased TS content (0.72 ± 0.05%) characterizes the presence of sulfur-containing components in plant tissue, including structural and metabolic compounds [44], which is typical for the East Kazakhstan region under conditions of sulfate salinity [45]. No direct data on the sulfur content in *S. europaea* were found for comparison, which underlines the originality of the results obtained.

Thus, the results of elemental composition analysis of the aboveground organs of *S. europaea* collected in East Kazakhstan showed the predominance of the organic carbon fraction, as well as the presence of nitrogen and sulfur in the analyzed sample. These data may reflect specific features of the biochemical composition of the plant growing in a saline habitat and provide additional information on the chemical characteristics of *S. europaea* from the regional flora.

GC-MS analysis of the ethanolic extract of the aboveground organs of *S. europaea* provided a tentative profile of lipophilic and semi-volatile compounds. This profile is generally consistent with previous data for *S. europaea* [46] and other species of the genus *Salicornia* [18,47]. Fatty acids and α-tocopherol were the main tentatively annotated constituents, while a fatty acid ester and several low-molecular-weight oxygenated compounds were also observed. The highest relative peak area was observed for linoleic acid, i.e., (9Z,12Z)-octadeca-9,12-dienoic acid (24.82%; NIST Qual = 99), indicating a notable contribution of unsaturated fatty acids to the GC-MS profile of the analyzed sample.

Other compounds with acceptable NIST library match quality and retention index agreement included α-tocopherol (vitamin E) (10.87%; NIST Qual = 97), hexadecanoic acid (palmitic acid) (7.34%; NIST Qual = 99), acetic acid (3.67%; NIST Qual = 80), and other minor compounds.

Overall, these data indicate the predominance of lipid-associated compounds in the GC-MS profile of the analyzed extract. The detection of linoleic and palmitic acids is consistent with the previously published data on the lipid composition of *S. europaea*, where these compounds have been described as characteristic constituents of the lipophilic fraction [18,48]. However, the relative abundance of individual compounds may vary. Such differences may be associated with the analyzed plant organ, growth conditions, extraction procedure, and chromatographic parameters. The α-tocopherol level detected in the analyzed sample was higher than the value reported by Karan et al. (2021) [1] for other populations of *S. europaea*.

It should be emphasized that the compounds were tentatively annotated based on comparison of their mass spectra with the NIST library. Authentic reference standards were not used. Therefore, the GC-MS results should be regarded as a characterization of the putative composition of the lipophilic and semi-volatile fraction of the analyzed sample rather than as definitive confirmation of individual compounds. Compounds with low NIST Qual values were not used to interpret the main patterns of the chemical profile and should be considered with caution.

The IR spectral profile of crude sample demonstrates a high concentration of organic compounds exhibiting osmotic and antioxidant activity. The presence of intense absorption bands of amide I and amide II indicates an active protein metabolism aimed at the synthesis of stress proteins. Also, the high intensity of the absorption bands at 1710 and 2923–2854 cm^−1^ indicates the predominance of lipid and pectin fractions, which contribute to the water retention effect under conditions of high salinity. The analysis of the ash residue indicates the presence of a carbonate-silicate matrix in the test sample, which confirms the biogenic accumulation of calcium and silicon compounds.

The FTIR spectroscopy results showed the presence of functional groups characteristic of proteins, lipids, pectic substances, and mineral components in the aboveground organs of *S. europaea* collected from a saline habitat in East Kazakhstan. These findings may reflect specific features of the biochemical composition of the plant associated with its adaptation to salinity conditions.

The metabolic profile of *S. europaea* is characterized by structural diversity and includes three main classes of secondary metabolites: benzoic acid derivatives, hydroxycinnamic acid derivatives, and flavonoids. The main phenolic pool is formed by derivatives of benzoic acid (vanillic acid, syringic acid, hydroxybenzoic acid) and hydroxycinnamic acids (ferulic acid, coumaric acid). The detection of glycoside and esterified derivatives, including vanillic acid hexoside, syringic acid hexoside, feruloyl hexoside, coumaroyl hexoside and coumaroylquinic acid, indicates the presence of soluble forms of phenolic compounds in the studied extract. Based on the literature data, such derivatives can be considered as a possible “soluble reserve” of phenolic metabolites, contributing to their accumulation, transport, and participation in maintaining plant redox homeostasis [49,50,51,52]. The identification of sinapic alcohol, as well as hydroxycinnamate derivatives, which, according to literature data, have photoprotective and antioxidant potential, may indirectly indicate the involvement of the phenylpropanoid pathway in the adaptive reactions of *S. europaea* [23,53]. Presumably, these compounds may be associated with cell wall strengthening, lignification, and protection of the photosynthetic apparatus, which is consistent with the known functions of phenolic metabolites in plants [54]. However, these conclusions should be considered as a biological interpretation based on the known functions of similar classes of metabolites, rather than as a direct experimental confirmation of the corresponding processes in the studied samples.

Flavonoid composition *S. europaea* exhibits high structural complexity, dominated by acylated derivatives of quercetin (quercetin-3-hexoside, quercetin-3-malonylhexoside, quercetin-3-acetylhexoside) and kaempferol-3-malonylhexoside. Malonyl and acetyl groups in flavonoid derivatives may modify their physicochemical properties, including stability and solubility [55].

All compounds were annotated at MSI level 2 (Metabolomics Standards Initiative) based on comparison of MS/MS spectra with library data and accurate *m*/*z* values with mass errors below 5 ppm [56]. The neutral loss of 162 Da observed for compounds 13–16, including quercetin-3-hexoside and its derivatives, indicates the presence of a hexoside moiety. The fragment ion at *m*/*z* 151 observed for the quercetin derivative may be associated with retro-Diels–Alder cleavage of the C-ring and further supports the annotation of a quercetin-type aglycone. For feruloylhexoside, the diagnostic fragments include *m*/*z* 193, corresponding to ferulic acid after the loss of a hexose residue, and *m*/*z* 134, associated with further fragmentation of the feruloyl moiety.

Thus, UPLC-HRMS/MS-based profiling indicated the presence of several classes of secondary metabolites in the analyzed sample, including hydroxycinnamic acid derivatives, benzoic acid derivatives, and flavonoids.

The obtained TPC values for *S. europaea* are consistent with those reported in [57], where the authors used 80% aqueous methanol for the extraction of phenolic compounds. According to [58], the extraction efficiency of phenolic compounds depends on the composition of the extractant, since a higher extraction capacity of the solvent may contribute to higher TPC values.

An analysis of the literature data on TFC and ABTS in *S. europaea* also shows the dependence of the flavonoid content on the stage of plant development, the extraction method, and the standard used for expressing results (QE and CE). Thus, in studies [58] using UAE for *S. europaea*, it was found that an increase in the proportion of ethanol in the extractant leads to an increase in the total content of flavonoids, with the maximum TFC values being ≈8–9 mg QE/g DW. The value of TFC obtained in this study is 2.40 ± 0.02 mg QE/g DW for an aqueous extract of aboveground organs. *S. europaea* confirms the presence of a flavonoid fraction in the raw material under study. At the same time, direct quantitative comparison of our data between other studies is limited due to the use of different standards (QE and CE), as well as differences in extraction conditions.

According to Limongelli et al. [59], the antioxidant activity of ABTS exhibits a pronounced dependence on extraction conditions and can vary in a wide range from 2.23 to 10.96 mg TE/g, depending on the solvent used. When using ultrasonic extraction with aqueous alcohol solutions, the antioxidant activity of ABTS can reach 21.44 ± 1.07 mg TE/g DW.

In the present study, ABTS radical-scavenging activity was observed together with moderate TPC and TFC values. This may be associated with the combined presence of phenolic compounds, flavonoids, and other bioactive components, including lipophilic compounds.

Vitamins A, C, and E are important biologically active compounds with pronounced antioxidant properties [60,61,62,63]. The studied sample is characterized by a moderate content of vitamins A, C, and E. The content of vitamin A significantly exceeds the literature data (0.01 mg/100 g), which may be due to differences in growing conditions and analysis methods [64]. The vitamin C content was close to the value reported by Evlash et al. [64] (1.25 mg/100 g DW), but remained low. The reason for the low vitamin C content may be a decrease in ascorbate synthesis in conditions of drought and high insolation, typical for Eastern Kazakhstan [65]. The vitamin E content in the studied sample falls within the range typical of other *Salicornia* species, such as *Salicornia ramosissima* [61]. At the same time, the level of α-tocopherol is dominant among the studied complex. The dominant role of α-tocopherol in antioxidant protection is confirmed by the ratio of vitamin E to β-carotenoid (≈3.4:1).

A limitation of the present study is the use of one composite biological sample collected from a single natural population. The repeated measurements reported as mean ± SD reflect only the variability of analytical measurements and do not show biological variability among individual plants, populations, or habitats. Therefore, the obtained results should be considered as a phytochemical characterization of the analyzed sample, rather than as evidence of population-level or regional variability in *S. europaea*.

## 4. Materials and Methods

### 4.1. Sampling and Sample Preparation

**Figure 5:**
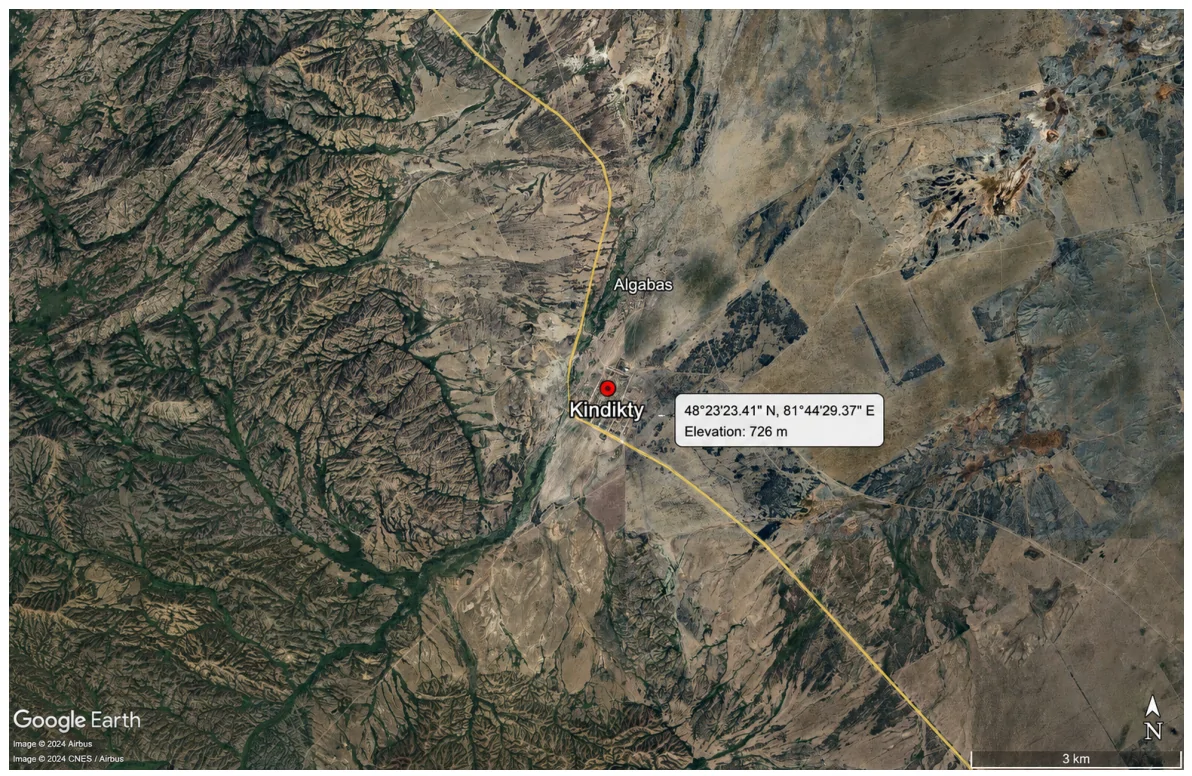
Location of the *S. europaea* sampling site near Kindikty, Abai Region, Kazakhstan (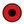
—the place of collection of plant material).

The aboveground organs of *S. europaea* were collected in September 2024 in the vicinity of the village of Kindikty (Aksuat district, Abai region) (Figure 5). The geographical coordinates of the sampling site were 48°23′23.41″ N and 81°44′29.37″ E, with an elevation of 726 m above sea level. The choice of this location is due to the need to obtain environmentally friendly plant raw materials that are not subject to man-made pollution.

The collected plant material was botanically identified as *S. europaea* based on morphological characteristics using regional floristic references, including Flora of Kazakhstan [9]. The taxonomic nomenclature was verified according to the Plants of the World Online (POWO) database [66]. The identification was confirmed by B.M. Silybayeva, Associate Professor at Alikhan Bokeikhan University, Semey, Kazakhstan. A voucher specimen was prepared and deposited in the Regional History and Local Lore Museum, Semey, Kazakhstan. No official voucher specimen number was assigned.

The selection criteria included the absence of visible mechanical damage and signs of phytopathologies. Plant material was collected from several visually healthy individuals within a single natural population over an approximately 10 × 10 m sampling area and combined to obtain one composite biological sample. After purification from impurities and pre-freezing, the collected raw materials were freeze-dried (SCIENTZ-12N Freeze Dryer, Ningbo Scientz Biotechnology Co., Ltd., Ningbo, China) at a temperature of −50 °C to a constant weight. The dried material was ground to a particle size of about 0.5–1 mm and stored in glass jars with sealed stoppers.

### 4.2. Organic Elemental Analysis

The content of total carbon (TC), organic carbon (TOC), inorganic carbon (TIC), total nitrogen (TN) and total sulfur (TS) was determined using a CNS Vario Max element analyzer (Elementar Analysensysteme, GmbH, Langenselbold, Germany) [29]. The samples were previously dried at 105 °C and ground in an agate Pulverisette 2 mill (Fritsch, GmbH, Idar-Oberstein, Germany).

TC [mass. %] was determined by catalytic combustion at 1140 °C, and TOC [mass %] was determined by thermal decomposition at 550 °C. The TIC content [mass %] was calculated from the difference between TC and TOC. All measurements were performed in three repetitions, followed by statistical processing.

### 4.3. Gas Chromatographic Mass Spectrometric Analysis

Gas chromatographic mass spectrometric analysis of ethanol extract of *S. europaea* was performed on an Agilent 7890A gas chromatograph with an Agilent 5975C mass-selective detector (Agilent Technologies, Inc., Santa Clara, CA, USA) [49]. For GC-MS analysis, the ethanolic extract was used and analyzed without derivatization. Separation was carried out on an Rtx-100DHA capillary column (30 m × 0.25 mm, film thickness 0.5 μm).

Analysis conditions: injector temperature—280 °C; thermostat mode: from 60 °C to 300 °C at a speed of 8 °C/min; ion source temperature—230 °C, and the quadrupole temperature was 150 °C. Helium was used as the carrier gas at a column pressure of 2 psi. The injection volume was 0.2 μL, and the sample was introduced in split mode with a split ratio of 20:1. The solvent delay was set to 1.5 min. Mass spectra were recorded in full-scan mode at a scan rate of 2.9 spectra/s. Compound annotation was performed by comparison of the obtained mass spectra with the NIST 08 mass spectral library. The total analysis time was 32 min. A homologous series of n-alkanes (C_8_–C_36_) was analyzed under the same chromatographic conditions. Experimental retention indices (RI_exp_) were calculated using the retention times of n-alkanes eluting immediately before and after each analyte.

The above-ground part of *S. europaea* was extracted in a Soxhlet apparatus using 90% C_2_H_5_OH (raw material: solvent ratio 1:20) at 80 °C for 6 h. The resulting extract was filtered, cooled, and then the solvent was removed on an IKA RV 10 rotary evaporator (IKA-Werke GmbH & Co. KG, Staufen, Germany) under vacuum.

### 4.4. IR-Fourier Spectroscopy

The FT-IR spectra of the feedstock and ash fraction were recorded in attenuated total reflection (ATR) mode using a Bruker ALPHA II spectrometer equipped with a diamond ATR module (Bruker Optics GmbH & Co. KG, Ettlingen, Germany) [67]. The measurements were carried out in the range of 4000–400 cm^−1^ with a resolution of 4 cm^−1^ (24 scans). A powder sample (≈10 mg) was placed on a diamond crystal and pressed with an integrated press. Before each measurement, the background spectrum of the air was recorded and purified with isopropanol.

The preparation of plant raw materials for IR spectroscopic analysis included freeze-drying for 24 h using an Alpha 1-2 LDplus freeze dryer (Martin Christ Gefriertrocknungsanlagen GmbH, Osterode am Harz, Germany) and grinding using an RM 200 mortar grinder (Retsch, GmbH, Haan, Germany). The resulting powder was divided into two parts: the first fraction (the initial sample) was used for IR analysis, the second fraction (ash) was mineralized in a Phoenix microwave muffle system (CEM Corporation, Matthews, NC, USA).

The samples were ozonized by stepwise heating: exposure at 250 °C (30 min), followed by an increase in temperature to 450 °C (50 °C step every 30 min). The total duration of the process was 5 h. To ensure statistical reliability, each sample was analyzed nine times (*n* = 9). Each IR spectrum was subjected to baseline correction and normalization using OPUS software, version 8.5 (Bruker Optics GmbH & Co. KG, Ettlingen, Germany). The characteristic bands were identified using the literature data.

### 4.5. Preparation of the Dry Extract for Phytochemical Analyses

The dry extract was used for UPLC-HRMS/MS, determination of TPC, TFC, antioxidant activity and vitamins. To obtain the dry methanolic extract, a 2.000 g portion of the sample, ground to a particle size of 0.5 mm, was placed in a Teflon vessel. Then, 45 mL of 70% methanol solution was added, and extraction was carried out in a microwave system MARS Xpress 5240 microwave system (CEM Corporation, Matthews, NC, USA). Extraction parameters: temperature—40 °C, power—800 W, intensity—100%. The resulting extract was filtered and quantitatively transferred to a round-bottomed flask. The extraction was repeated twice. The combined supernatants were quantitatively transferred to a measuring flask and the volume was adjusted with 70% methanol to 50 mL. The resulting extracted volume was evaporated dry on a rotary evaporator at a 40 °C, after which the remainder was weighed and the extract yield was calculated. The extraction yield was expressed as milligrams of extract obtained after solvent evaporation per 1 g of the dried mass of the sample used for extraction. Then, the dry extracts were dissolved in a 70% methanol solution to a concentration of 10 mg/mL, filtered using syringe filters with a nylon membrane and a pore size of 0.45 μm. The extract was stored in vials in the refrigerator at 4 °C before analysis, protecting them from light.

### 4.6. Ultra-Performance Liquid Chromatography—High-Resolution Mass Spectrometry (UPLC-HRMS/MS)

The extracts were analyzed using an ultra-performance liquid chromatography system (UPLC, Acquity, Waters) and hybrid quadrupole-orbital high-resolution mass spectrometry (HRMS/MS, Q-Exactive, Thermo Fisher Scientific GmbH, Bremen, Germany). The samples (5 µL each) were separated using an Acquity BEH Shield (2.1 × 150 mm, particle size 1.7 µm, water) at a temperature of 40 °C [68]. A mixture of water (component A, LC-MS grade, Merck KGaA, Darmstadt, Germany) and acetonitrile (component B, LC-MS grade, Merck KGaA, Darmstadt, Germany), each containing 0.1% formic acid, was used as the mobile phase. Elution was performed in a gradient mode (from 5% to 75% of component B). The flow rate was 0.3 mL/min.

Mass spectrometric analysis was performed in the negative ionization (ESI^−^) mode, which is suitable for phenolic compounds. Scanning range—*m*/*z* 150–1500, resolution 70,000. The source parameters were: capillary voltage 3.5 kV, source temperature 300 °C, desolvation temperature 350 °C, sheath gas flow 50 units. The initial UPLC-HRMS/MS data was processed using the FreeStyle software, version 1.8 SP2 (Thermo Fisher Scientific, Waltham, MA, USA). The analysis of the obtained data included the determination of accurate of *m*/*z* values, retention time, ionization mode, putative adducts and major MS/MS fragments for the preliminary annotation of metabolites. The compounds were tentatively annotated by comparing their MS/MS spectra with the MassBank and HMDB libraries. Criteria: mass error < 5 ppm and matching fragment ions. All compounds were annotated at the confidence level 2 according to the Metabolomics Standards Initiative (MSI). Fragmentation mechanisms in this mode include the loss of neutral fragments such as water (H_2_O, 18 Da), carbon dioxide (CO_2_, 44 Da) and monosaccharide units (162 Da for hexose, 132 Da for pentose). The reproducibility of the method was evaluated by three analytical replicates (*n* = 3) using the relative standard deviation (RSD) of retention time (<0.5%) and peak area (<15%). α-terpinol (10 μg/mL) was used as the internal standard.

### 4.7. Determination of Total Phenolic Content (TPC)

The total content of polyphenols was determined by the colorimetric method with the Folin-Chocalteu reagent [69]. Dilute Folin-Chocalteu reagent and 0.5 mL of 20% Na_2_CO_3_ solution were added to 100 μL of the methanolic solution. After incubation in the dark at room temperature for 2 h, the absorbance was measured at 760 nm (U-2810, Hitachi High-Tech Corporation, Tokyo, Japan). 

Calculations were performed using a gallic acid calibration curve (0.20; 0.16; 0.12; 0.08; 0.04 and 0.02 mg/mL). The results were expressed as milligrams of gallic acid equivalents per gram of dry plant raw material (mg GAE/g). All measurements were repeated three times (*n* = 3).

### 4.8. Determination of Total Flavonoid Content (TFC)

The total content of flavonoids was determined by the spectrophotometric method using aluminum nitrate [69]. A total of 1 mL of Al(NO_3_)_3_·9H_2_O solution was added to 1 mL of the methanolic extract solution (10 mg/mL). The reaction mixture was incubated for 5 min at room temperature. The absorbance was measured at 420 nm using a spectrophotometer (U-2810, Hitachi High-Tech Corporation, Tokyo, Japan), with background correction applied for colored extracts. Calculations were performed using a quercetin calibration curve over the concentration range of 0.015–0.150 mg/mL. The results were expressed as milligrams of quercetin equivalents per gram of dry plant raw material (mg QE/g). All measurements were performed three times.

### 4.9. Determination of Antioxidant Activity (ABTS Method)

Antioxidant activity was determined using the by the ABTS•^+^ radical cation decolorization assay [70]. The radical was obtained by mixing 14 mM solutions of ABTS (2,2′-azino-bis(3-ethylbenzothiazoline-6-sulfonic acid) and 7 mM K_2_S_2_O_8_ in equal volumes, followed by incubation in a dark place at room temperature for 12–20 h. The ABTS•^+^ working solution was diluted with deionized water to an absorbance of 0.70–0.80 at 734 nm. A total of 100 μL of diluted methanolic extract solution was added to 3.0 mL of the ABTS•^+^ working solution. Absorbance was measured at 734 nm using a spectrophotometer (U-2810, Hitachi High-Tech Corporation, Tokyo, Japan) after 6 min of incubation. Background correction was applied for colored samples.

The percentage inhibition of the ABTS radical was calculated using the following Equation (1):

(1) $$I,\%=\frac{A_{0}-A_{p}}{A_{0}}\times 100$$

where *I*—percentage of inhibition, *A*_0_—absorbance of the control sample, and *A_p_*—absorbance of the analyzed sample. The concentration of antioxidants was determined using a Trolox calibration curve (6-hydroxy-2,5,7,8-tetramethylchroman-2-carboxylic acid) over the concentration range of 0.02–0.20 mg/mL. Antioxidant activity was expressed as Trolox equivalents per gram of dry plant raw materials (mg TE/g). All measurements were repeated three times (*n* = 3).

## 5. Determination of Vitamin Content

The vitamin C content was determined using high-performance liquid chromatography (HPLC) on an Agilent 1200 Series system (Agilent Technologies, Inc., Santa Clara, CA, USA) [71]. Extraction was performed with a solution of metaphosphoric acid, and the total vitamin C content was determined after reduction in dehydroascorbic acid with L-cysteine. Separation was performed on a reversed-phase C18 column (250 × 4.0 mm, 5 µm) using a mixture of a solution of KH_2_PO_4_ and a solution of cetrimide in methanol (900:100, *v*/*v*) as the mobile phase. The flow rate was 0.7 mL/min, the injection volume was 30 µL, and detection was performed at 265 nm. Quantitative determination was performed using the external standard method.

The vitamin A content was determined after alkaline saponification of the sample with ethanolic KOH solution in the presence of an antioxidant, followed by extraction of the unsaponifiable fraction with an organic solvent. Chromatographic separation was performed on a normal phase column of LiChrosorb Si 60 (250 × 4 mm, 5 µm) using a mixture of *n*-hexane and *n*-butanol (98:2, *v*/*v*) as the mobile phase. Detection was performed at 325 nm. The quantitative content was determined according to the calibration schedule of standard all-*trans*-retinol solutions.

The vitamin E (α-tocopherol) content was determined by HPLC after preliminary alkaline saponification of the sample in the presence of an antioxidant and extraction with an organic solvent. The analysis was performed on a normal-phase silica gel column (250 × 4.6 mm, 5 μm) using a mixture of *n*-hexane and 2-propanol (99.5:0.5, *v*/*v*) as the mobile phase. Detection was carried out fluorimetrically at λ_ex_ 295 nm and λ_em_ 330 nm.

All measurements were carried out in three repetitions (*n* = 3), the results were expressed as mg/100 g of dry plant raw materials.

### Statistical Data Analysis

Statistical processing of the results of all experimental studies was performed using Microsoft Excel 2019 and OriginPro 2025 software (Origin, Version 2025. OriginLab Corporation, Northampton, MA, USA). All analytical measurements were carried out in triplicate (*n* = 3). The experimental data obtained are presented as an arithmetic mean ± standard deviation (M ± SD). Since the study was performed on a single biological sample, represented by a pooled plant sample collected from a single habitat, correlation analysis, analysis of variance (ANOVA) and multivariate methods (PCA, PLS-DA, HCA) were not applied. Descriptive statistics were calculated using the built-in software functions.

## 6. Conclusions

The conducted study provided a comprehensive phytochemical characterization of the aboveground organs of *S. europaea* collected from a saline habitat in Eastern Kazakhstan. The use of elemental analysis, GC-MS, FTIR spectroscopy, UPLC-HRMS/MS, and spectrophotometric methods made it possible to characterize the main groups of compounds in the analyzed sample.

Elemental analysis showed the predominance of the organic carbon fraction and revealed the presence of nitrogen and sulfur in the aboveground organs of *S. europaea*. These data characterize the elemental profile of the analyzed sample and may be associated with specific features of mineral nutrition and the chemical composition of the plant growing in a saline habitat. GC-MS analysis of the ethanol extract showed the presence of lipophilic and semi-volatile compounds. Among the tentatively annotated compounds with high library match scores, linoleic acid, hexadecanoic acid, and α-tocopherol were observed. These results characterize the lipophilic fraction of the studied plant material. FTIR spectroscopy complemented the chemical characterization of the analyzed sample by indicating the presence of functional groups associated with polysaccharides, proteins, lipids, and mineral components. UPLC-HRMS/MS profiling enabled the tentative annotation of secondary metabolites belonging to hydroxycinnamic acid derivatives, benzoic acid derivatives, and flavonoids.

Spectrophotometric analysis revealed the presence of polyphenols and flavonoids in the *S. europaea* extract and demonstrated its radical-scavenging activity under the ABTS assay conditions. In addition, the contents of vitamins A, C, and E were determined. Taken together, these results provide additional information on the bioactive composition of the aboveground organs of *S. europaea* from the regional flora.

It should be noted that the reported mean ± SD values reflect analytical variability within one composite biological sample and do not represent independent biological replication. Therefore, the obtained results cannot be generalized to all populations of *S. europaea* in Eastern Kazakhstan.

The obtained data allow *S. europaea* from Eastern Kazakhstan to be considered a promising object for further applied research. In future studies, these results may be used to investigate several natural populations, include independent biological replicates, analyze soil conditions, and assess the safety and biological activity of extracts in cell models and in vivo experiments.

## Figures and Tables

**Table 1:** GC mass spectrometric analysis data for ethanol extract of *S. europaea*.

Peak	Retention Time (min)	Area, %	Tentatively Annotated Compound/Library Name	IUPAC/Preferred Name	CAS No.	NIST Qual	RI_exp_	RI_lit_	Molecular Formula	M_W_
1	2.362	3.67	Acetic acid	Ethanoic acid	64-19-7	80	879	890	C_2_H_4_O_2_	60.05
2	2.725	1.04	1-hydroxy-2-Propanone	1-hydroxypropan-2-one	116-09-6	64	926	915	C_3_H_6_O_2_	74.08
3	12.924	2.69	Ethanone, 1-(3-methoxyphenyl)-	1-(3-methoxyphenyl)ethan-1-one	586-37-8	72	1350	1348	C_9_H_10_O_2_	150.17
4	22.430	7.34	n-Hexadecanoic acid	hexadecanoic acid	57-10-3	99	1962	1960	C_16_H_32_O_2_	256.42
5	24.476	24.82	9,12-Octadecadienoic acid (Z,Z)-	(9Z,12Z)-octadeca-9,12-dienoic acid	60-33-3	99	2130	2133	C_18_H_32_O_2_	280.4
6	24.828	0.98	Linoleic acid ethyl ester	ethyl (9Z,12Z)-octadeca-9,12-dienoate	544-35-4	64	2150	2148	C_20_H_36_O_2_	308.5
7	27.380	10.87	α-tocopherol (Vitamin E)	(2R)-2,5,7,8-tetramethyl-2-[(4R,8R)-4,8,12-trimethyltridecyl]-3,4-dihydro-2H-chromen-6-ol	59-02-9	97	2850	2855	C_29_H_50_O_2_	430.71

Note: NIST Qual indicates the quality of the mass spectral match with the NIST 08 library. RI_exp_—is the experimental retention index calculated using a homologous series of n-alkanes (C8–C36) analyzed under the same chromatographic conditions. RI_lit_—is the literature or database retention index value. The compounds were tentatively annotated based on the mass spectra, NIST Qual values, retention index agreement, and elution order. Since authentic reference standards were not used, all GC-MS annotations should be considered tentative.

**Table 2:** Characteristics of the main absorption bands in the IR spectra of *S. europaea* and its ash fraction.

Sample	Wave Number, cm^−1^	Type of Oscillation	Functional Group/Compound Type	References
Crude sample	3293	ν(O-Н) и ν(N-H)	-OН groups of polysaccharides (cellulose, hemicellulose), phenols; -NH group of amines	[28,29]
	2923–2854	ν_as_, ν_s_ (C-H)	CH_2_/–CH_3_ groups of lipids, waxes, and fatty acids	[28,30]
	1710	ν(C=O)	C=O groups of fatty acids and pectins	[31,32]
	1640	Amide I, ν(C=O)	Peptide bond, proteins	[33]
	1548	Amide II, ν(N-H), δ(C-N)	Proteins, amino acids	[31,32]
	1402	ν_s_(COO^−^)	Organic acid COO^−^ ions	[29]
	1322	δ (O-H) и δ(C-O)	Polysaccharides, components of amide III	[34]
	1141–1022	ν(C-O-C) и ν(C-O)	Glycoside bonds of cellulose and hemicellulose	[35]
Ash fraction	1480, 1441, 1427	ν(CO_3_^2−^)	Carbonates of alkaline earth metals	[36,37]
	1107, 1045	ν(Si-O-Si), ν(Si-O)	Metal silicates, SiO_2_	[36,38,39]
	620	δ(M–O)	Metal oxides and silicates	[40]

**Table 3:** Data from high-performance liquid chromatography of a sample of *S. europaea* aboveground organs.

№	RT [min]	Ion Mode	Adduct Ion	Observed *m*/*z*	Calculated *m*/*z*	Mass Error, ppm	Fragmentation	CE, eV	Display Formula	Putative Compound	Compound Class	Molecular Class, % of Detected Compounds	Level
1	3.40	ESI^−^	[M−H]^−^	301.0930	301.09179	4.17	121.0278, 139.0383	35	C_13_H_17_O_8_	Isotachioside	Benzoates	33.33	2
2	4.03	ESI^−^	[M−H]^−^	367.0648	367.06597	−3.31	137.0230, 181.3157, 261.6907	35	C_16_H_15_O_10_	Syringic acid hexoside	Benzoates	33.33	2
3	4.65	ESI^−^	[M−H]^−^	329.0879	329.08671	3.68	123.0437, 167.0338, 173.0286, 226.0909	35	C_14_H_17_O_9_	Vanillic acid hexoside	Benzoates	33.33	2
4	4.80	ESI^−^	[M−H]^−^	299.0771	299.07614	3.67	137.0230, 179.0341, 313.2883	35	C_13_H_15_O_8_	Hydroxybenzoic acid hexoside	Benzoates	33.33	2
5	5.45	ESI^−^	[M−H]^−^	179.0339	179.03389	0.28	134.9866, 151.0387, 179.0340	35	C_9_H_7_O_4_	Caffeic acids	HCA-derivatives	38.89	2
6	5.95	ESI^−^	[M−H]^−^	195.0651	195.06519	1.02	135.0434, 136.0518, 153.3114, 180.0419, 195.0657	35	C_10_H_11_O_4_	Ferulic acid	HCA-derivatives	38.89	2
7	6.12	ESI^−^	[M−H]^−^	283.0822	283.08123	3.48	137.0230, 171.8092, 239.0922	35	C_13_H_15_O_7_	Benzoic acid hexaside	Benzoates	33.33	2
8	6.43	ESI^−^	[M−H]^−^	355.1035	355.10236	3.34	134.0360, 193.0497, 217.0502, 355.1041	35	C_16_H_19_O_9_	Feruloyl hexoside	HCA-derivatives	38.89	2
9	6.57	ESI^−^	[M−H]^−^	325.0929	325.09179	3.48	145.0282, 163.0388, 265.0722, 325.1295	35	C_15_H_17_O_8_	Coumaroyl hexoside	HCA-derivatives	38.89	2
10	6.94	ESI^−^	[M−H]^−^	167.0337	167.03389	−0.89	108.0201, 123.0437, 152.0102, 167.0338	35	C_8_H_7_O_4_	Vanillic acid	Benzoates	33.33	2
11	7.93	ESI^−^	[M−H]^−^	337.0931	337.09179	3.81	163.0388, 191.0552	35	C_16_H_17_O_8_	Coumaroylquinic acid	HCA-derivatives	38.89	2
12	9.25	ESI^−^	[M−H]^−^	579.2084	579.20722	2.00	166.0260, 181.0496, 208.0365, 327.0515, 402.1319, 505.0917	35	C_28_H_35_O_13_	Syringaresinol hexoside	HCA-derivatives	38.89	2
13	9.65	ESI^−^	[M−H]^−^	463.0883	463.08710	2.56	151.0023, 271.0247, 300.0275, 301.0352, 313.1665	35	C_21_H_19_O_12_	Quercetin-3-hexoside	Flavonoids	27.78	2
14	9.67	ESI^−^	[M−H]^−^	301.0353	301.03428	3.42	177.4362, 183.1018, 201.1121, 269.0459, 285.0405, 286.0486, 301.2022	35	C_15_H_9_O_7_	Quercetin	Flavonoids	27.78	2
15	10.16	ESI^−^	[M−H]^−^	549.0888	549.08750	2.39	151.0024, 271.0249, 300.0276	35	C_24_H_21_O_15_	Quercetin-3-malonylhexoside	Flavonoids	27.78	2
16	10.17	ESI^−^	[M−H]^−^	505.0989	505.09767	2.52	151.0024, 271.0248, 300.0276, 301.0352, 343.0380, 463.0880, 505.0977	35	C_23_H_21_O_13_	Quercetin-3-acetylhexoside	Flavonoids	27.78	2
17	10.55	ESI^−^	[M−H]^−^	209.0813	209.08084	2.14	165.1275, 209.1187	35	C_11_H_13_O_4_	Sinapic alcohol	HCA-derivatives	38.89	2
18	10.96	ESI^−^	[M−H]^−^	533.0940	533.09258	2.65	284.0326, 285.0404, 341.0675	35	C_24_H_21_O_14_	Kaempferol-3-malonylhexoside	Flavonoids	27.78	2

Note: Class percentages were calculated based on the number of HPLC-detected compounds assigned to each class among the 18 compounds listed in Table 3.

**Table 4:** Quantitative content of vitamins in *S. europaea*.

Vitamin	Content, mg/100 g of Dry Matter
Vitamin A (β-carotene)	1.16 ± 0.11
Vitamin C (ascorbic acid)	1.27 ± 0.12
Vitamin E (α-tocopherol)	3.89 ± 0.38

Note: The data is presented as M ± SD (*n* = 3).

## Data Availability

The data presented in this study are available within the article.
